# The Critical Concentration of Nickel Sufficient for Growth and Nutrient Accumulation of Newhall Navel Orange

**DOI:** 10.3390/plants15121816

**Published:** 2026-06-12

**Authors:** Xiaojuan Wang, Chengxiao Hu, Qiling Tan, Songwei Wu

**Affiliations:** 1 Microelement Research Center, College of Resources and Environment, Huazhong Agricultural University, Wuhan 430070, China; wangxj67@webmail.hzau.edu.cn (X.W.); qltan@mail.hzau.edu.cn (Q.T.); wusw@mail.hzau.edu.cn (S.W.); 2 National Key Laboratory for Germplasm Innovation & Utilization of Horticultural Crops, Huazhong Agricultural University, Wuhan 430070, China

**Keywords:** Nickel, citrus, deficiency, excess, nutrients, accumulation

## Abstract

In citrus production, there is an absence of established standards of critical Nickel (Ni) content for deficiency, sufficiency, and excess, which could be used to determine the nutritional status of plant Ni. In this study, to explore the critical Ni concentrations for deficiency and excess, we conducted a hydroponic pot culture experiment and investigated the effects of Ni levels on flower and fruit development, dry weight, and nutrient accumulation of Newhall navel orange. We found that 0.8 and 6.4 mg L^−1^ of solution Ni were the turning point concentrations of Ni deficiency and excess for plants, respectively. Solution Ni deficiency (0 to 0.8 mg L^−1^ of Ni) tended to promote vegetative growth and increase the dry weight of new leaves, but suppress flower bud number and fruit development. It also significantly promoted the accumulation of N, P, K, Ca, and Mg in old leaves and N and K in roots, but significantly reduced that of Fe, Mn, and Zn in roots. Excess solution Ni (6.4 to 12.8 mg L^−1^ of Ni) reduced the water content of fruit peel and was accompanied by fruit cracking during the fruit expansion period, inhibited new leaf growth and whole plant biomass or dry weight, and significantly decreased nutrient accumulation in roots. Equations of dry weight and solution Ni levels for each plant organ were established, showing that 3.93 to 4.72 mg L^−1^ of Ni was the sufficient concentration of solution Ni for the growth and development of Newhall navel orange, with the corresponding range of Ni contents in new and old leaves being 17.87 to 20.42 and 10.24 to 11.64 mg kg^−1^, respectively. These findings provide reference for the recommended range of Ni sufficient for citrus growth.

## 1. Introduction

Nickel (Ni) is the 17th micronutrient element that was most recently identified as essential for plant growth and development. In 1987, Brown found that Ni application would affect barley seed germination, root elongation, nitrogen (N) metabolism, and iron (Fe) absorption. Ni deficiency would also cause barley to fail in normal growth, which proved that Ni is a necessary nutrient element for better plant growth [1]. An appropriate concentration of Ni plays a significant role in promoting plant biomass, N metabolism, and plant disease resistance [2], especially in N metabolism, which significantly increases crop yields [3]. Plants exhibit a relatively low demand for Ni, with Ni concentrations in common plant species ranging from 0.05 to 10 mg kg^−1^ [4]. Notably, the symptoms of Ni deficiency are analogous to those of copper (Cu) and zinc (Zn) deficiency [5], and as a result, Ni is rarely supplemented intentionally in practical agricultural production.

Ni is inherently a metallic element, driven by both natural processes and anthropogenic activities, and Ni ions cycle through the atmosphere, soil, and aquatic environments within ecosystems [6]. Elevated Ni levels have been documented across global regions, including North America, Asia, and Europe. Ni concentrations in aquatic and terrestrial resources are 0.2 mg L^−1^ and 26 g kg^−1^, respectively—approximately 25-fold higher than those in unpolluted resources [7]. According to the Bulletin on the National Soil Pollution Survey jointly released by the Ministry of Ecology and Environment and the Ministry of Natural Resources of China, Ni ranks as a primary heavy metal pollutant in cultivated land, grassland, and unused land, second only to cadmium (Cd) [8].

Associated with metal pollution driven by rapid industrial development in southern China, an assessment of Ni content in food and dietary exposure risk revealed that foods with elevated Ni levels are predominantly concentrated in economically developed regions of southern China [9]. Notably, China’s citrus cultivation is primarily distributed in southern regions, with Fujian Province, Jiangxi Province, and Hubei Province serving as major production areas [10]. Field surveys have been carried out to determine Ni concentrations in 51 citrus orchards across four provinces in southern China. The soil available Ni concentrations and pulp Ni concentrations ranged primarily from 0.1 to 1.5 mg kg^−1^, while leaf Ni concentrations spanned 2.0 to 8.0 mg kg^−1^—all of which exhibited significant correlations with fruit quality indices [11]. Due to the absence of established standards for critical Ni content in citrus, it remains infeasible to assess whether the Ni concentrations observed in orchards fall within deficient or excessive ranges.

China leads the world in both citrus cultivation area and production volume [12]. In citrus orchards, the overapplication of macronutrient fertilizers and insufficient levels of meso- and micronutrients are prevalent issues. Accordingly, it has been proposed to reduce the application of N, phosphorus (P), and potassium (K) fertilizers, while balancing and increasing the use of meso- and micronutrient fertilizers [13]. The application of boron (B)- and molybdenum (Mo)-containing fertilizers has been shown to enhance the yield and quality of Wogan/Orah (*Citrus reticulata* ‘Wo Gan’) and Wenzhou mandarin (*Citrus reticulata* Blanco cv. Unshiu), as well as increase the content of macro- and micronutrients in the pulp [14]. The application of meso- and micronutrient fertilizers can mitigate the adverse effects associated with the overuse of N, P, and K fertilizers, sustain simplified and efficient fertilization practices, and improve fruit yield and quality metrics. However, research on the correlation between Ni and citrus nutrient status is scarce, and its specific role remains unclear.

Therefore, we hypothesized that there exists an optimal range of leaf Ni concentration in Newhall navel orange trees, within which plant growth and nutrient accumulation are better, while both Ni deficiency and excess would impair biomass production and disturb nutrient homeostasis. We employed Newhall navel orange as the experimental material in this study to investigate the effects of Ni levels on citrus tree growth, development, and nutrient accumulation. The objectives were to analyze the impacts of Ni deficiency and excess on citrus growth and development, provide a scientific basis for recommending the optimal range of Ni concentrations in citrus leaves, and offer theoretical support for the practical application of Ni fertilizers in citrus production.

## 2. Results

### 2.1. Effect of Ni Levels on Growth and Development of Newhall Navel Orange

**Figure 1:**
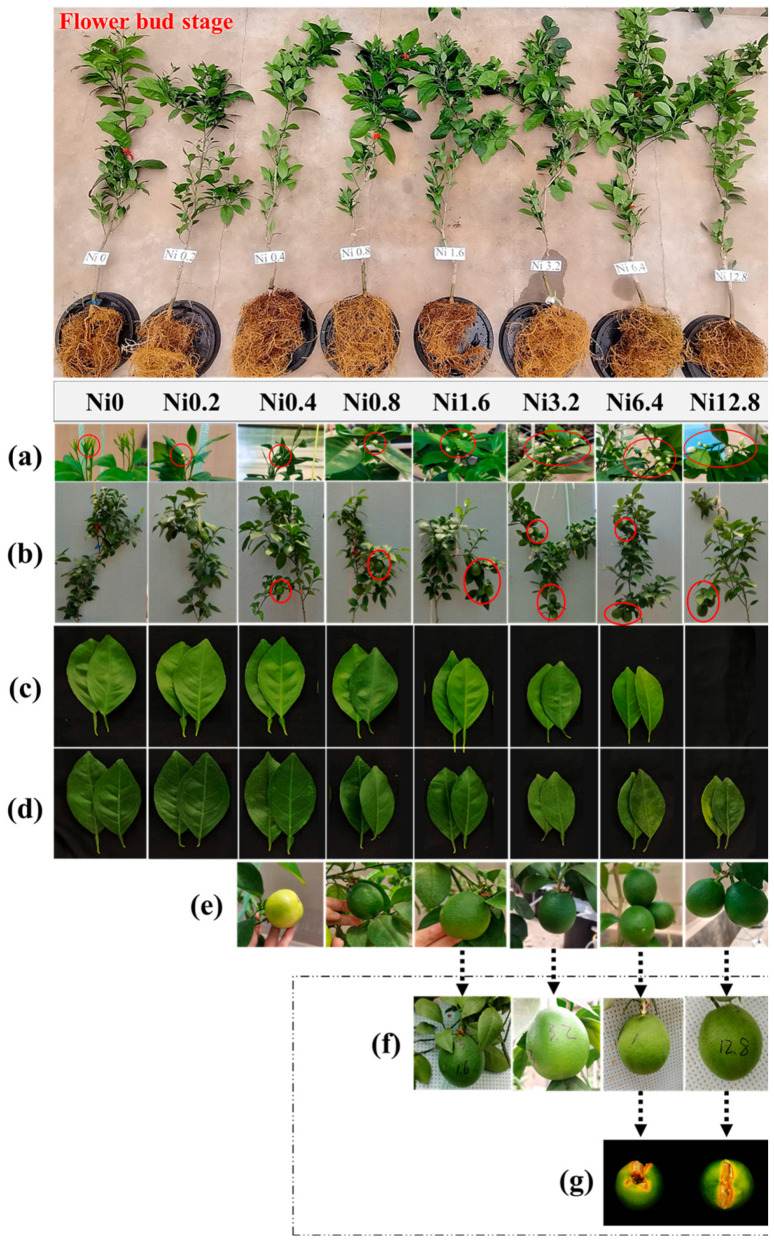
Effects of Ni on growth and development of Newhall navel orange tree. (**a**) flower bud, (**b**) fruit drop stage, (**c**) new leaves (expansion stage), (**d**) old leaves (expansion stage), (**e**,**f**) fruit, (**g**) fruit cracking (expansion stage). Note: the red circle indicates the labeled fruit in (**b**), the numbers on the fruits in (**f**) represent the corresponding Ni levels.

At the flowering stage, no bud for Ni0 and more than three buds for Ni1.6 to Ni12.8 indicated that Ni application improved the number of flowers (Figure 1a). At the fruit development stage, no fruit setting occurred for Ni0 and Ni0.2 (Figure 1b), fruit drop for Ni0.4 and Ni0.8 and fruit cracking for Ni6.4 and Ni12.8 took place (Figure 1e–g), and normal fruit occurred only for Ni1.6 and Ni3.2, showing that sufficient Ni application improved fruit development, but Ni deficiency or excess resulted in fruit drop or cracking, respectively. Compared to new and old leaves across all Ni treatments, only Ni12.8 exhibited no new leaf development, meaning that excess Ni inhibited the development of new leaves (Figure 1c,d). The results indicated that 0 to 0.8 mg L^−1^ of Ni inhibited flower and fruit development, while 6.4 to 12.8 mg L^−1^ of Ni inhibited new leaf development and caused fruit peel cracking.

### 2.2. Effect of Ni Levels on Dry Weight of Newhall Navel Orange

**Figure 2:**
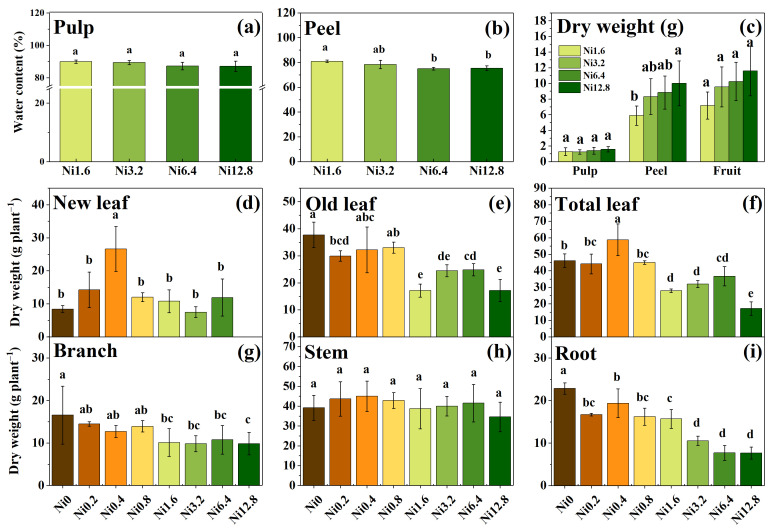
Effect of Ni on dry weight of various organs in Newhall navel orange. (**a**) pulp water content, (**b**) peel water content, (**c**) Fruit dry weight, (**d**–**i**) dry weight of various organs). Note: (**d**–**i**) includes four additional color bar graphs compared to (**a**–**c**), representing the dry weight of Ni0–Ni0.4. Values of all parameters are shown as means ± standard deviation. Different lowercase letters represent significant differences among Ni treatments at the same stage by Duncan’s test (*p* < 0.05).

Compared with Ni1.6, the water content of the peel decreased significantly by 6.00% and 5.57% at Ni6.4 and Ni12.8, respectively (Figure 2b), and there was no significant difference in the water content and dry weight of the pulp (Figure 2a,c). The water content of the peel decreased during continuous fruit development, which was speculated to be the cause of fruit cracking. Compared with Ni0, the dry weight of old leaves, total leaves (total dry weight of new and old leaves), and branches at Ni1.6 decreased significantly by 54.57%, 39.49%, and 38.91%, respectively, and reached the lowest values at Ni12.8 (Figure 2e–g). Compared with Ni0, the dry weight of roots began to significantly decrease at Ni0.2 and was reduced by 31.29% at Ni12.8 (Figure 2i). The dry weight of leaves at Ni0 to Ni0.8 was significantly higher than that at Ni1.6 to Ni12.8 (Figure 2f). The results indicated that 0 to 0.8 mg·L^−1^ of Ni could promote vegetative growth (leaves and branches), while 6.4 to 12.8 mg·L^−1^ of Ni inhibited reproductive growth (fruit).

### 2.3. Ni Content and Accumulation in Various Organs of Newhall Navel Orange

**Figure 3:**
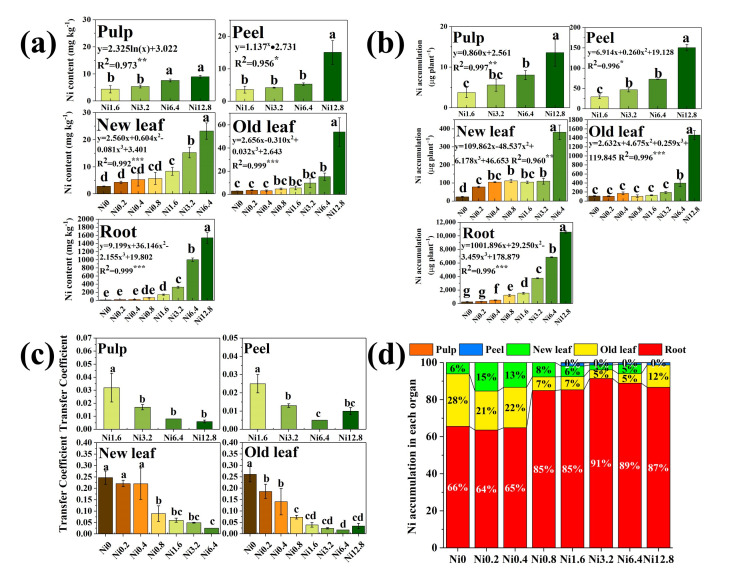
Effect of Ni levels on the Ni content and accumulation of various organs in Newhall navel orange. (**a**) Ni content (mg kg^−1^ DW), (**b**) Ni accumulation (μg tree^−1^ DW), (**c**) Ni transfer coefficient, (**d**) Ni accumulation distribution (%). The equations represent the linear fitting equations of nutrient solution Ni levels with Ni content and accumulation in different organs. *, **, and *** superscripts after R^2^ values indicate statistical differences at *p* < 0.05, <0.01, and <0.001, respectively. Values of all parameters are shown as means ± standard deviation. Different lowercase letters (a, b, c…) represent significant differences determined by Duncan’s test (*p* < 0.05).

Ni content and accumulation in each organ of the Newhall navel orange gradually increased with Ni levels. Compared with Ni0, Ni content and accumulation in the pulp showed significant differences at Ni6.4, while the peel showed significant differences at Ni12.8 and Ni3.2, respectively. New leaves also showed the same at Ni1.6 and Ni0.2, respectively, and those in the old leaves showed the same at Ni6.4. Ni content and accumulation in the root showed significant differences at Ni1.6 and Ni0.4, with R^2^ values of 0.999 (*p* < 0.001) and 0.996 (*p* < 0.001), respectively (Figure 3a,b). The goodness of fit and equation correlation were better than those of other organs, indicating that the root was the most sensitive organ to Ni concentration. The Ni translocation coefficient in the new and old leaves showed significant differences at Ni0.8 and Ni6.4 (Figure 3c), and the proportion of Ni accumulation began to be significantly enriched in the roots at Ni0.8 (Figure 3d), suggesting that Ni0.8 was the turning point concentration for Ni absorption, distribution, and accumulation. Below Ni0.8, Ni in the roots was translocated to the aboveground parts, while that from Ni6.4 to Ni12.8 was reduced and accumulated in roots.

### 2.4. Effect of Ni Levels on Nutrient Accumulation in Newhall Navel Orange

As shown in Table 1, Mn accumulation in the pulp is significantly negatively correlated (*p* < 0.01) with Ni levels. The accumulation of all nutrients in the old leaves is negatively correlated with Ni levels, among which N, P, K, Mg, and Mn show an extremely significant negative correlation (*p* < 0.001). The N, K, Ca, and Mg in the root show an extremely significant negative correlation (*p* < 0.001) with Ni levels, while Mn shows a significant positive correlation (*p* < 0.05). This indicates that nutrient accumulation in the leaves and root is significantly inhibited with the increase in Ni concentration, and Mn is the nutrient most affected by Ni.

**Figure 4:**
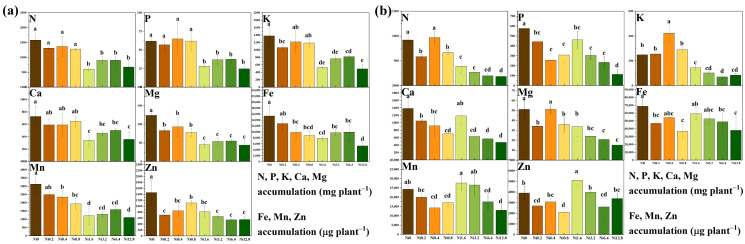
Effects of Ni levels on nutrient accumulation in old leaves (**a**) and roots (**b**) of Newhall navel orange. Values of all parameters are shown as means ± standard deviations. Different lowercase letters (a, b, c…) represent significant differences determined by Duncan’s test (*p* < 0.05).

The Ni concentration at the turning point of nutrient accumulation in old leaves and roots is also 0.8 mg L^−1^ of Ni (Figure 4). The accumulation of Fe and Mn in old leaves significantly and gradually decreased from Ni0 to Ni0.8 (Figure 4a); that of P, Ca, Fe, Mn, and Zn in roots significantly and gradually decreased from Ni0 to Ni0.8; that of nutrients decreased gradually from Ni1.6 to Ni12.8 (Figure 4b) and decreased with the reduction in root dry weight from Ni0 to Ni12.8; and that of Fe, Mn, and Zn significantly increased from Ni1.6 to Ni3.2 (Figure 4b). These results indicate that nutrient accumulation in leaves and roots decreases with the reduction in Ni levels, while sufficient Ni (1.6 to 3.2 mg L^−1^ Ni) promotes Fe, Mn, and Zn accumulation in roots.

### 2.5. Optimal Ni Concentration in Nutrient Solution and Leaf Ni Content for Newhall Navel Orange

**Figure 5:**
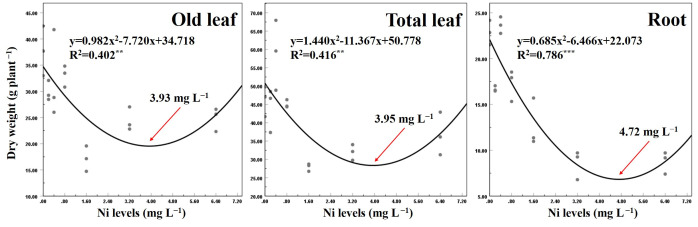
Regression fitting of the dry weight and Ni level of each organ of Newhall navel orange. The equations represent the linear fitting equations of nutrient solution Ni levels with dry weight in different organs, N = 24. **, and *** superscripts after R^2^ values indicate statistical differences at *p* < 0.05, <0.01, and <0.001, respectively.

Regression analysis was performed to examine the relationship between Ni levels and the dry weight of each organ. Significant correlations were found in old leaves, total leaves, and roots (those without significant correlations are not listed) (Figure 5). Under the condition of normal fruit development, the dry weight of old leaves, total leaves, and roots began to increase when the Ni concentration in the nutrient solution was 3.93, 3.95, and 4.72 mg L^−1^, respectively. This indicates that theoretically, the range of Ni concentrations of 3.93 to 4.72 mg L^−1^ is optimal for vegetative and reproductive growth of Newhall navel orange. Substituting this range of concentrations into the equations for leaf Ni content (Figure 3a), the Ni content in new leaves was 17.87–20.42 mg kg^−1^, and 10.24–11.64 mg kg^−1^ in old leaves.

## 3. Discussion

### 3.1. Effects of Ni Deficiency on the Growth, Development, and Nutrient Accumulation of Newhall Navel Orange

In citrus production, tree regulation measures are often adopted to preserve flowers and fruits. Specifically, by removing vigorous branches and shoots to reduce the consumption of ineffective nutrients, nutrients are allocated to flowers and fruits. Vegetative growth competes with reproductive growth in crops, and if flower and fruit development is poor, nutrients will shift towards vegetative growth. In this study, Ni deficiency inhibited tree growth before flower bud differentiation, resulting in a significant reduction in the number of flower buds (Figure 1). Therefore, in the later stage, due to the absence of competition for nutrients from fruit growth and development, the tree experienced intensive growth, and the dry weight of new leaves and roots both increased significantly (Figure 2). This phenomenon can be ascribed to nutrient imbalance.

**Figure 6:**
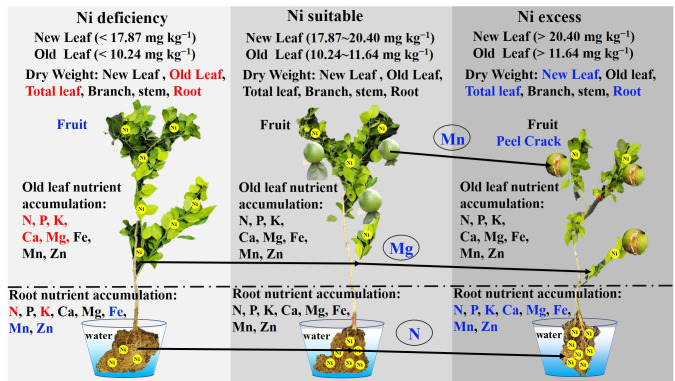
Effects of Ni deficiency and excess on the growth and nutrient accumulation of Newhall navel orange. The yellow background tint represents the distribution of Ni. Red font indicates significant promotion of the index compared with sufficient Ni, blue font indicates significant inhibition, and black font indicates no significant difference.

A lower N content in the citrus tree is more conducive to flowering, while an excessive amount may inhibit flower bud differentiation. Applying high-concentration N fertilizer before flower bud differentiation will stimulate the citrus to produce a high number of new shoots, consuming the nutrients of the tree, and thus significantly reducing the number of flowers in the next season [15]. Therefore, C/N ratio theory suggests that a low ratio of carbohydrate to N accumulation can promote flower bud differentiation in plants—high carbohydrate provides an energy basis for flower bud differentiation, while low N levels reduce the competition for nutrients between vegetative and reproductive growth [16]. In citrus cultivation and management, girdling is often used to reduce the N content in the tree, promote soluble sugar accumulation, and thereby increase the C/N ratio of leaves [17,18]. However, fruit expansion is one of the peak periods when citrus trees require N. Sufficient N promotes the division and expansion of fruit cells, laying the foundation for large fruit formation, and facilitating the transport of photosynthetic products to the fruits, thereby enhancing the fruit setting rate [19]. In this study, N accumulation in leaves and roots significantly increased at Ni deficiency (Figure 6), indicating that Ni deficiency interfered with the N metabolism of citrus trees.

Ni deficiency in plants can affect the core functions of N metabolism, thereby triggering complex metabolic disorders [20]. The reduction in urease activity leads to a decline in N reuse efficiency, with N being fixed in an ineffective form; an increase in inorganic N and a decrease in organic N; and an inability to efficiently synthesize proteins. At the same time, the disorder in organic acid metabolism results in insufficient energy supply for C metabolism, leading to a C/N imbalance. Crops are unable to efficiently utilize the absorbed N and cannot provide the energy and material basis for reproductive growth [21]. Under such circumstances, the branches of Newhall navel orange do not have sufficient organic N to synthesize proteins and thus lack the ability to become fruit-bearing mother branches. Additionally, N metabolism products (such as glutamic acid) are precursors for the synthesis of various hormones [22]. During the flower bud differentiation period, Ni deficiency may indirectly disrupt the endogenous hormone balance and interfere with the transition from vegetative to reproductive growth.

The increase in dry weight of leaves and roots can simultaneously enhance nutrient accumulation. However, in this study, we observed significant differences in trace elements, with the accumulation of Mn and Zn in roots showing a particularly significant decline (Figure 4b). It is speculated that the core mechanism of this phenomenon is related to interaction and absorption regulation among nutrients. Previous studies have shown that, in citrus production, K and Zn have antagonistic effects, and when the K content is too high, it can easily induce Fe and Zn deficiency [23]. In this study, N and K accumulation in roots significantly increased, which might indirectly inhibit Fe, Mn, and Zn accumulation by competing for absorption sites or inhibiting the activity of transport proteins.

Additionally, Ni can upregulate the gene expression of iron-regulated transporter 1 (IRT1) as a key channel for the root absorption of divalent metal ions such as Fe^2+^, Zn^2+^, and Mn^2+^, and its increased expression can enhance the absorption efficiency of trace elements [24,25]. The Ni concentration needs to be maintained within an appropriate range; Ni excess can inhibit the absorption of other nutrients through antagonistic effects, while Ni sufficiency can not only enhance the transport protein activity to increase the net absorption of nutrients [26], but also promote overall growth and metabolic activity by participating in plant N metabolism, providing sufficient energy and carbon skeletons for root absorption, and indirectly strengthening the absorption and assimilation process of trace elements. In conclusion, compared with Ni sufficiency, the reduction in the accumulation of trace elements in the roots under Ni deficiency is the result of the combined effects of nutrient antagonism, transport protein regulation, and insufficient metabolic support.

### 3.2. Effects of Ni Excess on the Growth, Development, and Nutrient Accumulation in Newhall Navel Orange

The results of this study indicate that Ni excess has a significant impact on the reproductive growth of Newhall navel orange. When Ni accumulation during the flower bud stage does not reach the toxic threshold, there are no abnormalities in plant growth and development. However, as the growth period progresses to the fruit expansion stage, symptoms of Ni stress become significantly evident on the fruits, specifically manifested as a decrease in fruit peel water content accompanied by cracking (Figure 1g).

The stress symptoms caused by Ni excess are similar to those of other heavy metals, with the core mechanism being damage to the cell membrane system, which subsequently leads to disorders in plant growth and morphological parameters, imbalances in nutrient dynamics, alterations in physiological and biochemical characteristics, and oxidative stress [27,28,29]. Previous studies have confirmed that Ni excess can affect plant growth through pathways such as inhibiting root and stem growth and biomass accumulation, reducing plant water content, interfering with N metabolism, suppressing key enzyme activities, inducing lipid peroxidation, and reducing seed vigor [8,30]. For instance, in soybeans under Ni excess conditions, biomass significantly decreases, and the CO_2_ assimilation rate, stomatal conductance, and transpiration rate of leaves all significantly decline simultaneously [31]. Ni toxicity repressed photosynthesis and interfered with micronutrient accumulation in tomato seedlings, reduced the concentrations of auxin, cytokinin and gibberellic acid, and altered the expression of genes involved in carbon/nitrogen metabolism pathways [32]. In addition, barley reprograms its carbon and nitrogen metabolic networks to enhance dark fixation, the TCA cycle, the pentose phosphate pathway, and the nitrogen assimilation enzyme system, in order to cope with the increased energy demand and Ni stress [6]. In this study, excess Ni suppressed the growth of new leaves, fruit cracking, root dry weight, and nutrient accumulation, all of which are typical responses of plants to Ni stress, with the significant reduction in root biomass being particularly prominent.

As the core organ for nutrient absorption in plants, the root system can absorb and isolate excessive Ni, thereby triggering multiple stress responses. On one hand, Ni excess can interfere with and inhibit the function of specific auxin transport proteins (PIN2) in the root meristem. On the other hand, it can induce a significant accumulation of reactive oxygen species (ROS) within the roots, damaging the integrity of cell structure and function, and ultimately leading to the shedding of lateral roots and the loss of their physiological functions [6,30]. Plants possess active defense mechanisms to regulate Ni toxicity. In *Arabidopsis thaliana*, Ni excess can induce the Fe transporter AtIREG2 in the roots to isolate excessive Ni ions into the vacuoles of root cells [33]. Additionally, organic acids such as citric acid and histidine can bind with Ni to form chelates and be stored in the vacuoles [34]. This “vacuolar sequestration” mechanism effectively inhibits the excessive migration of Ni from the roots to the aboveground parts, making the roots the main “storage site” for Ni and thereby protecting the normal development of aboveground organs. Based on these mechanisms, during the fruit growth and development period, nutrient accumulation in the old leaves of the aboveground parts under Ni excess showed no significant difference from that under Ni sufficiency, but the nutrient accumulation in the roots was significantly reduced (Figure 6c). This result confirms the protective effect of the roots on the aboveground parts; the roots maintain the nutrient supply to the aboveground parts by retaining Ni excess and sacrificing their own nutrient accumulation.

Mn (pulp) and Mg (old leaves) accumulation was extremely significantly negatively correlated with Ni levels (Table 1). Mn is an essential element for maintaining the structure and function of chloroplasts and directly participates in the water photolysis reaction of photosynthesis [35]. Mg is a core component of chlorophyll, which directly determines the photosynthetic efficiency of leaves [36]. The significant reduction in accumulation of both elements indicates that Ni excess has a significant inhibitory effect on the photosynthesis of Newhall navel oranges. Previous studies have pointed out that fruit expansion is the peak period of Mg demand, and the Mg content in leaves is prone to decline during this period, requiring timely supplementation of Mg fertilizer [37]. In this study, Mg accumulation in old leaves was extremely significantly negatively correlated with Ni levels, which is speculated to be related to the high demand for Mg by the plant during the expansion period. At the same time, Ni excess interferes with nutrient absorption by the root system, further exacerbating the imbalance between Mg supply and demand. In addition, the core physiological process during the fruit expansion period is water absorption by cells for expansion, and Ni excess inhibits nutrient absorption by roots (especially Mn), which can lead to disorders in water metabolism and ultimately cause fruit cracking.

### 3.3. Ni Content in Leaves That Is Sufficient for the Growth and Development of Newhall Navel Orange

The average Ni content in an adult human body is approximately 6–10 mg [38,39]. Animal model studies have demonstrated that dietary Ni concentrations ≥ 250 μg g^−1^ induce signs of Ni toxicity [40]. In 2020, the European Food Safety Authority (EFSA) set the Tolerable Daily Intake (TDI) of Ni for nickel-sensitized humans at 13 µg/kg bw/day, representing the maximum safe daily intake of Ni from food and drinking water for an adult [41]. “bw” stands for body weight, indicating that an individual’s upper limit of safe intake varies with their weight. For instance, a 60-kg adult should not consume more than 780 µg of Ni per day (60 kg × 13 µg/kg = 780 µg). In the present study, the optimal Ni concentration in nutrient solution for growth and development was determined to range from 3.93 to 4.72 mg L^−1^. Within this range, Ni accumulation in pulp was 5.94 to 6.62 μg per fruit. Using the maximum pulp Ni accumulation (6.62 μg per fruit) for calculation (780 µg ÷ 6.62 μg ≈ 117.82), an adult weighing 60 kg needs to theoretically consume at least 117 Newhall navel oranges every day to reach the EFSA TDI. However, humans do not typically consume such large quantities of citrus daily. Additionally, not all ingested Ni is bioavailable—excess Ni is excreted via the skin, urine, and hair [42,43]. Thus, the optimal Ni content identified in this study is theoretically appropriate and safe for both fruit development and human consumption.

A field survey of background Ni levels in the primary Newhall navel orange production region (Ganzhou City, Jiangxi Province, China) showed that leaf Ni concentrations across all surveyed orchards ranged from 1.74 to 12.47 mg kg^−1^ (mean: 4.77 mg kg^−1^), while pulp Ni concentrations ranged from 0.25 to 1.19 mg kg^−1^ (mean: 0.70 mg kg^−1^) [11]. Notably, these ranges are lower than the minimum Ni concentrations observed in this study (10.24 mg kg^−1^ in old leaves and 7.16 mg kg^−1^ in pulp). In 2023, findings from a study investigating the response of perennial ryegrass to Ni supplementation demonstrated that the majority, or even an overwhelming majority, of soils may derive benefits from Ni addition. This observation suggests that crops cultivated in the soils of Wisconsin and Illinois, USA, could potentially encounter Ni deficiency [44]. These findings suggest that current Ni levels in commercial crop cultivation environments (e.g., citrus orchards) may be deficient. Therefore, further field trials are warranted to validate whether Ni concentrations in citrus orchards are indeed suboptimal under actual production conditions.

## 4. Materials and Methods

### 4.1. Plant Materials and Growth Conditions

The Newhall navel orange trees used in the experiment were provided by Hubei Green Care Agricultural Technology Co., Ltd (Wuhan, China). The grafting rootstock used was Poncirus trifoliata (trifoliate orange); the experiment was conducted in the Huazhong Agricultural University National Citrus Breeding Center Seedling Greenhouse in Wuhan County, Hubei Province.

These 3-year-old Newhall navel orange trees (*Citrus sinensis* Osbeck cv. ‘Newhall’) were cleaned with water and grown in buckets for 15 days with only deionized water (ensuring the seedlings consumed the original nutrients). After that, deionized water was replaced with an 8 L nutrient solution with an electromagnetic air pump with 24 h of aeration at 40 L min^−1^ (ensuring the roots have enough oxygen) for each bucket, and the nutrient solution was replaced every 7 days. The nutrient solution was modified according to Hoagland and Arnon: Ca (NO_3_)_2_·4H_2_O 4 mmol L^−1^, KNO_3_ 6 mmol L^−1^, NH_4_H_2_PO_4_ 1 mmol L^−1^, MgSO_4_7H_2_O 2 mmol L^−1^, H_3_BO_3_ 46.2 µmol L^−1^, MnCl_2_·4H_2_O 9.1 µmol L^−1^, ZnSO_4_·7H_2_O 0.8 µmol L^−1^, CuSO_4_·5H_2_O 0.3 µmol L^−1^, (NH_4_)_6_Mo_7_O_24_·4H_2_O 0.1 µmol L^−1^, and Fe-Na EDTA 100 µmol L^−1^; pH = 5.8–6.0.

### 4.2. Experimental Design

Eight treatments were designed, including the control and seven treatment groups (Ni0, Ni0.2, Ni0.4, Ni0.8, Ni1.6, Ni3.2, Ni6.4, and Ni12.8), applying NiSO_4_·6H_2_O at 0, 0.2, 0.4, 0.8, 1.6, 3.2, 6.4, and 12.8 mg L^−1^, respectively. Each treatment consisted of four replications. After 10 months of cultivation, from the growth stage to the fruit expansion period, fruits showed Ni stress symptoms at Ni6.4 to Ni12.8. In order to ensure a sufficient sample, the plants were harvested at the fruit expansion stage, and fruit (pulp and peel), leaf (new and old), stem, branch, and root samples were collected.

### 4.3. Sample and Sample Preparation

Forty disease-free leaves (20 pieces each of new and old leaves) were collected from each plant and cleaned sequentially with 0.1% neutral detergent, clean water, 0.2% dilute nitric acid solution for 30 s, and deionized water for 2 min. The samples were dried at 105 °C for 30 min and at 65 °C until constant weight, and then ground into powder with an agate mortar.

The fruit samples were divided into two parts (pulp and peel); peel samples were cleaned sequentially with 0.1% neutral detergent, tap water, 0.2% dilute nitric acid solution for 30 s, and deionized water for 2 min. Pulp and peel were dried at 105 °C for 30 min and at 65 °C until constant weight, and then ground into powder with an agate mortar. A quarter of the root samples were collected from each seedling, with the same preparation as the leaves.

### 4.4. Measurements and Analysis

The nutrient content in leaves is expressed on a dry weight basis [45]. The dried and ground samples were treated with the H_2_SO_4_-H_2_O_2_ digestion method. The N content was determined by distillation titration, P content by the molybdenum antimony anti-colorimetric method, and K content by the flame photometer method. The dried and ground samples were digested with HNO_3_-HClO_4_ (*v*/*v* = 20:1) to determine the Ni content by ICP-MS (Juguang Technology Co., Ltd., Hangzhou, China), and digested with HNO_3_-HClO_4_ (*v*/*v* = 9:1) to determine the Fe, Mn, Zn, Ca, and Mg contents by using an atomic absorption spectrophotometer (Z 2000, HITACHI, Tokyo, Japan). Each sample was analyzed in triplicate, and the mean value was used. The quality control standard sample is “GBW10014a (GSB-5a) Cabbage,” produced by the Institute of Geophysical and Geochemical Exploration, Chinese Academy of Geological Sciences.

The fresh weight (FW) and dry weight (DW) of all samples were determined. Water content (%) = (FW − DW)/FW × 100. Nutrient accumulation (μg or mg) = nutrient content (mg kg^−1^ or g kg^−1^) × DW (g).

### 4.5. Statistical Analysis

SPSS 25.0 analytical software was used to perform statistical analysis on the data. Values are expressed as mean ± standard deviation (SD). Significance testing was conducted using Duncan’s multiple range test at a significance level of *p* < 0.05. Graphics were produced using Origin 2021.

## 5. Conclusions

In our study, we investigated the effects of Ni levels on the vegetative and reproductive growth of Newhall navel orange through long-term observations over the developmental period. Ni deficiency increased N accumulation in the trees and significantly promoted vegetative growth, while inhibiting flower bud differentiation and fruit development and reducing trace element accumulation in roots. Excess Ni supply led to decreased tree biomass and fruit cracking, and a significant reduction in nutrient accumulation in roots. Based on tree biomass and fruit development indicators, we recommend the following optimal Ni content ranges for Newhall navel orange: 17.87–20.42 and 10.24–11.64 mg·kg^−1^ in new and old leaves, respectively. It is expected that future research will focus on conducting verification trials in citrus orchards to provide a reference for the scientific application of Ni fertilizers in actual citrus production.

## Figures and Tables

**Table 1:** Correlation between Ni levels and nutrient accumulation of various organs in Newhall navel orange.

Pulp			Old Leaf		Root	
Coefficient		R^2^	Coefficient	R^2^	Coefficient	R^2^
N	-	-	−0.430	0.427 ***	−0.584	0.727 ***
P	-	-	−0.395	0.453 ***	−0.239	0.410 **
K	-	-	−0.364	0.431 ***	−0.556	0.624 ***
Ca	-	-	−0.293	0.241 *	−0.399	0.452 ***
Mg	-	-	−0.513	0.548 ***	−0.522	0.717 ***
Fe	-	-	−0.183	0.273 *	-	-
Mn	−0.653	0.510 **	−0.463	0.449 ***	0.170	0.178 *
Zn	-	-	−0.321	0.263 *	-	-

Note: The coefficient represents the b value of the quadratic equation, and R^2^ is the adjusted value. *, **, and *** superscripts after R^2^ values indicate statistical differences at *p* < 0.05, <0.01, and <0.001, respectively, N = 24. Nutrient accumulation in the peel and new leaves had no significant correlation with Ni levels and thus are not listed. - indicates no significant correlation.

## Data Availability

The original contributions presented in this study are included in the article. Further inquiries can be directed to the corresponding author.
